# Progressive encephalopathy due to chronic exposure to lead

**DOI:** 10.1055/s-0042-1758393

**Published:** 2022-12-19

**Authors:** Bárbara Cristina Vieira de Aquino, Agábio Diógenes Pessoa Neto, Mariana Galvão de Lima Martins Freire, Emanuela Coriolano Fidelix, Paulo Santiago de Morais Brito, Manuel Moreira Neto, Rodrigo Alencar e Silva, Clécio de Oliveira Godeiro Junior

**Affiliations:** 1 Hospital Universitário Onofre Lopes, Departamento de Neurologia, Natal RN, Brazil.; 2 Hospital Universitário Onofre Lopes, Departamento de Radiologia, Natal RN, Brazil.

**Figure 1:**
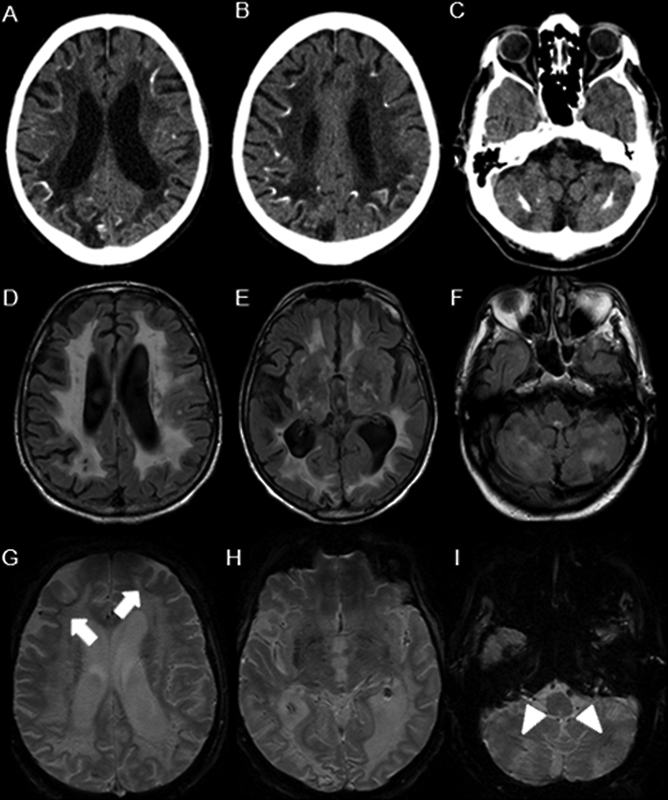
Axial non-contrast computed tomography (CT) scan of the brain showing diffuse hypodensity in the deep white matter of both cerebral hemispheres with cerebellar and cerebral subcortical calcifications (A-C). Axial T2/fluid-attenuated inversion recovery (FLAIR) magnetic resonance imaging (MRI) scan showing confluent hypersignal in the periventricular, subcortical, and deep white matter of both cerebral hemispheres (D), the thalamus (E), and the cerebellar hemispheres (F), with hypointense areas involving the basal ganglia (E). Axial gradient echo (GRE) T2-weighted magnetic resonance imaging (MRI) scan showing subcortical white matter (G: arrows) with either microbleeds or mineralization in the basal ganglia (H) and cerebellar hemispheres (I: arrowheads).

A 70-year-old man reported a 2-year history of progressive gait imbalance and cognitive decline (memory, executive functions, and language impairment). A physical examination revealed spastic ataxia without peripheral involvement. The patient worked with car battery solutions for 40 years, until 13 years ago. 1 He had several hospitalizations due to acute lead poisoning, with levels of lead in the blood and urinary delta-aminolevulinic acid of 162,8 g/dl and 20 mg/U (normal: up to 40 g/dl and 4,5 mg/U) respectively). Recent brain neuroimages showed typical findings of saturnism ( Figure 1 ), 2 with normal bone profile blood tests. Therefore, chronic lead poisoning should be remembered as an environmental cause of leukoencephalopathy. 2 3 4 5
